# Supplementary material for: A locally-blazed ant trail achieves efficient collective navigation despite limited information
Source: eLife. 2016 Nov 5;5:e20185. doi: 10.7554/eLife.20185 (PMC5142812; doi:10.7554/eLife.20185)
Supplement: Supplementary file 1. — DOI: http://dx.doi.org/10.7554/eLife.20185.021 [file elife-20185-supp1.pdf]

1 Probabilistic Following Noisy Local Advice (PF-NLA)

In this section we present our navigation model on graphs, called *Probabilistic Following* of *Noisy Local Advice (PF-NLA)*, which is a special case of the very general Random Walk in Random Environment model (see Section 2). The PF-NLA model is composed of two intertwined components, the underlying *Noisy Local Advice* component which provides unreliable navigation instructions (road signs), and the *Probabilistic Following* component which describes the responsiveness of the navigating entity to these navigation instructions. We first start with some basic definitions.

Let $G = (V, E)$ be an unweighted undirected graph over n nodes, where V represents the set of nodes of G and E represents the set of edges. Let $\tau \in V$ be a specified *target* destination node. Let $\texttt{dist}(\cdot,\cdot)$ be the distance function relative to G, that is, $\texttt{dist}(x, y) = k$ if the shortest path from x to y in G contains precisely k edges. The set of neighbors of each node x is divided into *correct* neighbors and *incorrect* neighbors. Specifically, correct neighbors of x are those closer to τ than x, or in other words, y is a correct neighbor of x if $\texttt{dist}(y, \tau) = \texttt{dist}(x, \tau) - 1$. All other neighbors of x are called incorrect.

A *mobile agent* A is initially placed on one of the nodes $\sigma \in V$ and aims at reaching the destination node τ by traversing the edges of G. Each traversal of an edge takes one unit of time. The goal of the agent is to reach τ as fast as possible as a function of $d = \texttt{dist}(\sigma, \tau)$, its initial distance to the target. A-priori, the agent has no knowledge of where the target node is and may furthermore have very limited knowledge regarding the structure of the graph G. At any time during its navigation, the agent may use guiding information held by its current hosting node. This guiding information, termed *advice*, is randomly chosen with a certain bias towards the correct direction of movement, as described below.

Noisy Local Advice. The model is specified by a *mistake*-parameter $\mu \in [0, 1]$. Each node $x \in V$ is assigned an advice $a(x)$ that is a pointer pointing to one of x's neighbors in G. Specifically, with probability $1 - \mu$, and independently of all other nodes, the advice $a(x)$ is *correct* and otherwise it is *incorrect*. Correct advice at x points at a correct neighbor of x, chosen uniformly at random among the correct neighbors of x. Similarly, incorrect advice at x points at an arbitrary incorrect neighbor of x, chosen uniformly at random among the incorrect neighbors of x.

Remark 1 (Reliability parameter)**.** *Note that in the numerical simulations, we use a slightly different convention: with probability* $0 < p < 1$*, called the* reliability parameter*, the advice* $a(x)$ *at each node* x *points at a correct neighbor of* x *(chosen uniformly at random among the correct neighbors), and otherwise, with probability* $1 - p$*, it points at an arbitrary neighbor of* x*, chosen uniformly at random among* all *neighbors of* x *(in contrast to choosing it only among incorrect neighbors). While this convention is slightly more intuitive than the convention that uses the language of the mistake-parameter* μ*, the latter is slightly more general as is also allows for the case in which the majority of advice is incorrect.*

The parameter μ governs the unreliability of the advice. Specifically, when $\mu = 0$, all advice is completely reliable, as it always leads toward a node on the shortest path to τ. In this case,

the strategy of always following the advice results in the agent reaching the target node τ in the shortest possible time, namely, d. Note, however, that when $\mu > 0$, the strategy of always following the advice can be detrimental by creating deadlocks. For example, on the 1-dimensional line graph, if there is one node with incorrect advice on the way to the target node, then an algorithm that always listens to advice will never reach the target. On the other extremity, when $\mu = 1$, the advice at any node becomes useless, and no strategy employed by the agent can allow for short traversal time even on very simple graphs.

The Probabilistic Following (PF) algorithm. Both extreme strategies, of always following the advice, or always ignoring it, fail when advice is unreliable. Instead, we suggest the basic strategy of following the advice with some fixed probability and ignoring it otherwise. Specifically, the *Probabilistic Following (PF)* algorithm is specified by a *listening*-parameter λ, where $0 \leq \lambda \leq 1$. At any given time t during the execution and for any node x, if the agent is at x at time t, then the neighbor y of x to be visited on the next hop is chosen at random, as follows:

- With probability λ, the next neighbor y of x to visit is the neighbor specified by the advice $a(x)$.
- With probability $1 - \lambda$, the neighbor y is chosen uniformly at random among x's neighbors.

Note that PF is a *memoryless* algorithm. That is, the decision at each node x regarding the next hop is based only on the current advice at x. In particular, no information from the agent's history is available to the algorithm.

Remark 2 (Concerning continuous models)**.** *The noisy local advice model assumes that each node has a single pointer advice. An alternative could consider that each node has several advice pointers at different strengths, pointing at different neighbors. In such cases, one would need to specify how the bias to the correct direction is manifested. One example could be that the strongest pointer is guaranteed to point at a correct direction with probability at least* $1 - \mu$. *This model can be reduced to our noisy local advice model, by considering the strongest advice as the single advice of the node. In general, one may view our model as a discretization of such models, and as a first approximation to them.*

Remark 3 (Concerning worst-case adversarial models)**.** *The model of navigation with advice was introduced in [4] and further studied in [3]. The main difference between the model of [3, 4] and our model is that in the model therein, the nodes holding incorrect advice (called* liars *in the terminology of [3, 4]) are chosen by an* adversary*, while in our model, which is better suited as a biologically driven model, they are chosen independently at random. This difference between the worst case placement of liars as assumed in [3, 4] and the average case scenario we use here is, in fact, substantial. For example, on the line graph our PF algorithm executed in the underlying NLA model can handle a linear number of nodes with incorrect advice and still reach the destination in linear time. In contrast, in the adversarial model of [3, 4], a worst-case placement of a linear number of liars will result in exponential hitting time for any memoryless algorithm.*

2 The model of Random Walks in Random Environments (RWRE)

In this section we describe the general model of Random Walks in Random Environments (RWRE). For more information, see the surveys [2, 5].

Consider a graph G. Informally, an *environment* ω is a distribution at each node x, indicating, for each neighbor y of x, the probability $\omega_x(y)$ of moving from x to y. That is, whenever the particle is at x at some time t, it will move to y at the next hop with probability $\omega_x(y)$. The given environment ω is chosen at random. That is, there is a governing distribution $\mathcal{P}_x$ at each node x, from which the distributions at x are sampled. The movement of the agent (typically called *particle* in the terminology of RWRE) is then analyzed taking expectation over the environments ω and walks in those environments. Note that once an environment ω is fixed, the movement of the particle is Markovian. However, without conditioning on the environment, the movement in not Markovian — this is a major source of technical difficulties in the analysis of RWRE.

We next provide a formal definition of the RWRE model. Our exposition is based on the surveys [2, 5]. For the sake of simplicity we will omit measurability related questions.

2.1 Definitions and notations

Consider a graph $G = (V, E)$. We denote by $x, y, z \ldots \in V$ vertices of G. We denote by $\texttt{dist}$ the graph distance on G and by ∂_A the boundary of a set $A \subseteq V$. In symbols:

$$\partial_A = \{x : \texttt{dist}(x, A) = 1\}$$

In this section G will in fact be restricted[1] to be the grid $\mathbb{Z}^d$ for some $d \in \mathbb{N}$, but we define the model on a general graph in anticipation of what we want to do next.

For each $x \in V$, let $\mathcal{P}_x$ denote a set of probability distributions supported on ∂_x. In other words, for each distribution in $\mathcal{P}_x$ only neighbors of x have positive mass.

Definition 4 (environment)**.** *An* environment ω *is an element of the* environment space $\Omega = (\mathcal{P}_x)_{x \in V}$*. In other words, an environment $\omega = (\omega_x)_{x \in V}$ is a collection of probability laws indexed by $x \in V$. For each $x \in V$, ω_x is a probability distribution over the neighbors of x.*

Definition 5 (random walk in the environment ω)**.** *Let $\omega \in \Omega$ be an environment, and assume that a particle is initially located at x. We denote by $(X_t)_{t \in \mathbb{N}}$ the Markov Chain on G whose transitions are given by ω. More precisely:*

$$P_{\omega,x}(X_0 = x) = 1, \tag{1}$$

$$P_{\omega,x}(X_{t+1} = y' \mid X_t = y) = \omega_y(y'). \tag{2}$$

and we call it the random walk in random environment ω started at x*. In particular, note that entails that $\sum_{y'} \omega_y(y') = 1$.*

[1]Although RWRE can apply for any graph, the literature on this subject has been focusing mostly on grid graphs of different dimensions.

So far we only have one source of randomness, $P_{\omega,x}$. To complete the model of RWRE, it remains to specify a law for the environment. We denote by $\mathbb{P}$ a measure on Ω. In words, $\mathbb{P}$ corresponds to averaging over the environment, and $P_{\omega,x}$ to averaging over the walk, conditioning on the environment. Overloading notation, we also write $\mathbb{P}_x$ in place of $\mathbb{P} \times P_{\omega,x}$. Thus $\mathbb{P}_x$ corresponds to averaging both over the environment and the walk. [2] As mentioned, one of the difficulties arising in the study of RWRE is that under $\mathbb{P}_x$, the walk is generally no longer Markovian. We will write $E_{x,\omega}$ to denote expectation under $P_{\omega,x}$, and $\mathbb{E}_x$ to denote expectation under $\mathbb{P}_x$.

We will further make two standard assumptions on the law of the environment.

2.1.1 Assumptions

(IID) Under $\mathbb{P}$, the distributions $(\omega_x)_{x\in V}$ are independent (and identically distributed if the graph G is the grid).

(EC) There exists a constant $\kappa > 0$ such that:

$$\mathbb{P}\left(\min_{(x,y)\in E(G)} \omega_x(y) \geq \kappa\right) = 1.$$

We call the maximal constant κ appearing in (EC) the *ellipticity constant.* This condition implies that the probability to move from every node to any of its neighbors is bounded from below by κ.

2.2 The PF-NLA model as a sub-model of RWRE

The Probabilistic Following Noisy Local Advice (PF-NLA) model is a special case of RWRE. Specifically, given the underlying setting of NLA with mistake-parameter μ and the PF algorithm with listening-parameter λ, the walk of the agent is specified as an instance of RWRE defined as follows.

A graph G is given, together with a collection of advice $(a_x)_{x\in V}$. For each $x \in V$, recall that $a_x \in V$ is some neighbor of x which we think of as a recommendation. If x is " correct" $\texttt{dist}(a_x, \tau) = \texttt{dist}(x, \tau) - 1$. Given the collection of advice $(a_x)_{x\in V}$ for each point x, we define the environment ω as the collection of transition probabilities $(\omega_x)_{x\in V}$, defined as follows.

- $\omega_x(a_x) = \lambda + (1-\lambda)/\Delta_x$, where Δ_x denotes the number of neighbors of x.
- $\omega_x(z) = (1-\lambda)/\Delta_x$, for every other neighbor $z \neq a_x$ of x.

Note that in the PF-NLA model, the environment ω_x defined above corresponds to the transition probabilities of the agent at x that executes PF, given the advice a_x. The law according to which each environment ω_x is chosen is then specified by the law of the advice a_x, as given by the underlying NLA setting.

[2]In the RWRE literature, $P_{\omega,x}$ is often referred to as the *quenched* law of the walk, and $\mathbb{P}_x$ as the *averaged* or *annealed* law.

3 Results on the line

In this section, we consider the line graph $H = 0, 1, \ldots, d$. The agent starts at the source node $\sigma = 0$ and wishes to reach the target node $\tau = d$. The agent executes PF with listening-parameter λ over an underlying NLA setting with mistake-parameter μ. The following theorem characterizes the conditions that allow linear expected hitting time.

Theorem 6. *For any mistake-parameter $\mu < 1/2$, there is a $C > 0$ (which depends on μ only) such that for any fixed listening-parameter $\lambda \in (0, 1 - 2\mu)$ guarantees the expected hitting time is less than $C \cdot d$.*

Remark 7. *By essentially the same proof, we would get that the same result holds even if H is infinite to the left. We emphasize that the advice gives an advantage with respect to simple random walk as a simple random walk would only get a hitting time of d^2 to reach d from 0 on a finite line and ∞ to reach d from 0 on the infinite line.*

To prove the theorem, we adapt known arguments and results from the theory of RWRE on the line to the context of our advice setting. For this purpose, we view the PF-NLA as a sub-model of RWRE, see Section 2.2. A PF-NLA model with a mistake-parameter μ and a listening-parameter $0 < \lambda < 1$ corresponds to a random environment ω for the line. For any $x \in H$ let us denote by $q_x = \omega(x, x-1)$ and $p_x = \omega(x, x+1)$. We also define the following important notion as in [7].

Definition 8. [**governing ratio**] *For any point x, the governing ratio of x is $\rho_x = \frac{q_x}{p_x}$.*

Note that the expectation $\mathbb{E}(\rho_x)$ is the same for all x. Therefore, for convenience we term this value as $\mathbb{E}(\rho)$ and call it the *governing ratio.* Solomon [6] proved that for the infinite line, if $\mathbb{E}(\rho) < 1$ then the speed of the walker is positive and equal to $\frac{1-\mathbb{E}(\rho)}{1+\mathbb{E}(\rho)}$ (going to $+\infty$). In Lemma 9 we show that the same result holds on the finite case and in Lemma 11 we show that if the mistake-parameter $\mu < 1/2$, then there exists a listening-parameter $0 < \lambda < 1$ such that the resulting PF-NLA model has $\mathbb{E}(\rho) < 1$. Combining these two lemmas we obtain the theorem.

Lemma 9. *Consider a random walk on a random environment on the line graph H. The random environment is summarized through the parameter $\alpha := \mathbb{E}(\rho)$.*

- *If $\alpha < 1$ then the expected time to hit d is linear,* i.e., *of the form $c(\alpha)d$ for some function $c(\cdot)$.*
- *If $\alpha > 1$ then the expected time is exponential in d, i.e. equivalent to $c(\alpha)\alpha^d$ when $d \to \infty$ for some function $c(\cdot)$.*
- *Else, if $\alpha = 1$ it is quadratic (in d).*

To prove Lemma 9, we use a convenient phrasing of a standard formula (see Proposition 3 in Chapter 5 of [1]). For completeness, we also give a proof of that formula, since it is rather short.

Lemma 10. *Consider a birth and death chain on $[0, 1 \dots, d]$ specified by the parameters $(q_x, p_x)_{x\in[d]}$ (which collectively correspond to an environment ω following our terminology). Then:*

$$E_{0,\omega}(T(d)) = d + 2 \sum_{i<j\leq d} \prod_{x=i}^{j} \rho_x.$$

For $i \in [d]$, let $\theta_i := E_{i,\omega}(T(i+1))$ be the expected time to reach $i+1$ starting at i. In expectation, $1/p_j - 1 = \rho_j$ excursions from j are needed before moving to $j+1$ and the length of one such excursion is $1 + \theta_{j-1}$ so it easily follows that, for $j \geq 1$

$$\theta_j = (1 + \theta_{j-1}) \cdot \rho_j + 1,$$

which yields

$$\theta_j = 2 \sum_{i\leq j} \prod_{i\leq x\leq j} \rho_x + 1,$$

and we get the formula by using $E_{0,\omega}(T(d)) = \sum_{j=0}^{d-1} \theta_j$. This completes the proof of Lemma 10.

Proof: [Proof of Theorem 6] We use the formula in Lemma 10 to prove Lemma 9. Under the (IID) assumption (see Section 2.1.1), we have that:

$$\mathbb{E}_0(T(d)) = \mathbb{E}E_{0,\omega}(T(d)) = d + 2 \sum_{j=1}^{d} \prod_{x=i}^{j} \mathbb{E}(\rho_x). \tag{3}$$

If we write $\alpha := \mathbb{E}(\rho)$, we get that:

$$\mathbb{E}_0(T(d)) = d + 2 \sum_{j=1}^{d} \sum_{i=0}^{j} \alpha^{j-i} = d + 2 \sum_{k=1}^{d} (d-k)\alpha^k. \tag{4}$$

If $\alpha < 1$ then Equation 4 translates to

$$\mathbb{E}_0(T(d)) \leq d(1 + 2 \sum_{k\geq 1} \alpha^k) = d\left(\frac{1+\alpha}{1-\alpha}\right),$$

which proves the first part of Lemma 9.

Also, Equation 4 states that $\mathbb{E}_0(T(d)) > \alpha^{d-1}$, which is exponential in d if $\alpha > 1$. Let us compute more precisely the asymptotics when $d \to \infty$, using Equation 4.

$$\begin{aligned}\sum_{k=1}^{d} (d-k)\alpha^k &= d\frac{\alpha - \alpha^{d+1}}{1-\alpha} - \alpha\left[\frac{\alpha - \alpha^{d+1}}{1-\alpha}\right]' \\ &= \alpha^{d+1}\frac{1}{(1-\alpha)^2} + d\frac{\alpha}{1-\alpha} + \frac{\alpha}{(1-\alpha)^2}. \end{aligned} \tag{5}$$

We see that the leading term is an exponential in d with base α and the second one a linear term in d. This proves the second part of Lemma 9, with $c(\alpha) = \frac{1}{(1-\alpha)^2}$. Finally, if $\alpha = 1$ then Equation 4 states that $\mathbb{E}_0(T(d)) = \Theta(d^2)$. This completes the proof of Lemma 9.

Theorem 6 will follow by combining Lemma 9 with the following lemma.

Lemma 11. *If the mistake-parameter $\mu < 1/2$, for any choice of $\lambda < 1 - 2\mu$, the resulting PF-NLA model has $\mathbb{E}(\rho) < 1$*

To prove the lemma, consider some listening-parameter $0 < \lambda < 1$. Observe first that the governing ratio $\rho = q/p$ satisfies: $\rho = \frac{1-\lambda}{1+\lambda}$ with probability $1-\mu$ and $\rho = \frac{1+\lambda}{1-\lambda}$ with probability μ. Hence, the expected value of the governing ratio is:

$$\mathbb{E}(\rho) = \frac{1-\lambda}{1+\lambda}(1-\mu) + \frac{1+\lambda}{1-\lambda}\mu. \tag{6}$$

It follows that:

$$\begin{aligned}
& \mathbb{E}(\rho) < 1 \\
\Leftrightarrow \quad & \mu\left(\frac{1+\lambda}{1-\lambda} - \frac{1-\lambda}{1+\lambda}\right) < 1 - \frac{1-\lambda}{1+\lambda} \\
\Leftrightarrow \quad & \mu\left(\frac{4\lambda}{(1+\lambda)(1-\lambda)}\right) < \frac{2\lambda}{1+\lambda} \\
\Leftrightarrow \quad & \mu < \frac{1-\lambda}{2} \\
\Leftrightarrow \quad & \lambda < 1 - 2\mu\ .
\end{aligned}$$

Hence, setting the listening-parameter λ to any positive number strictly less than $1-2\mu$ yields expected hitting time $O(d)$. This completes the proof of Lemma 11 and Theorem 6. □

The formula from Lemma 10 used in our context allows to compute the optimal choice of λ for any given μ, as we show in the following proposition.

Proposition 12. *Given a mistake-parameter $\mu < 1/2$, the listening-parameter λ^*, which minimizes the expected hitting time of the target, satisfies*

$$\frac{1-\lambda^*}{1+\lambda^*} = \sqrt{\frac{\mu}{1-\mu}}\ .$$

$$\lambda^* = \frac{1-\sqrt{\frac{\mu}{1-\mu}}}{1+\sqrt{\frac{\mu}{1-\mu}}}$$

Proof: As the expression in Equation (4) shows, the speed is decreasing with $\mathbb{E}(\rho)$ (recall that $\alpha = \mathbb{E}(\rho)$ in that expression). We thus maximize the *speed* by minimizing $\mathbb{E}(\rho)$. As expressed in Equation 6, for a fixed μ the optimal listening-parameter λ is given by minimizing:

$$\frac{1-\lambda}{1+\lambda}(1-\mu) + \frac{1+\lambda}{1-\lambda}\mu\ .$$

We differentiate w.r.t. λ to find that the optimal choice λ^* is such that:

$$\frac{-2}{(1+\lambda^*)^2}(1-\mu) + \frac{2}{(1-\lambda^*)^2}\mu = 0,$$

which implies that the best algorithm listens with probability λ^* satisfying: $\frac{1-\lambda^*}{1+\lambda^*} = \sqrt{\frac{\mu}{1-\mu}}$. In other words, we have:

$$\lambda^* = \frac{1-\sqrt{\frac{\mu}{1-\mu}}}{1+\sqrt{\frac{\mu}{1-\mu}}} \ .$$

□

Remark 13. The case of correlated advice. *Consider again the line graph H, and divide it to intervals of length k, namely: $[0, k-1]$, $[k, 2k-1]$, etc. (for simplicity assume that $d-1$ is divisible by k). Now consider the case that all advice on each interval are simultaneously either wrong (with probability μ) or correct (with probability $1-\mu$), independently of all other intervals. An agent that executes PF with any constant listening parameter $\lambda > 0$, would then need to spend $\exp(\Omega(k))$ expected time to pass each wrong interval in the correct direction. Since there are a linear number of wrong intervals in expectation for each positive mistake parameter μ, we get that any non-trivial PF algorithm will require expected hitting time of $d\exp(\Omega(k))$. This shows that many areas with correlated bad advice, can be devastating for the performance of PF.*

4 Results on the d-dimensional grid

In this section, we consider a variant of our PF-NLA model on the d-dimensional infinite grid $\mathbb{Z}^d$. In this variant, which is more consistent with the literature on RWRE, there is no particular target node and instead the mobile agent is interested in going to the "east". At any point x, the correct neighbor of x is the one to its "right" (i.e., pointing to the east) and all other neighbors are incorrect. In addition, we do not evaluate performances by the hitting time, and instead we are concerned with the *speed* of the agent, as defined in the following theorem.

Theorem 14. *Let $\delta > 0$ be a fixed parameter. Consider the PF-NLA model on the d-dimensional infinite grid $\mathbb{Z}^d$. There exists a sufficiently small mistake-probability $0 < \mu < 1$ (dependent on d), such that for any choice of listening parameter in the PF model $\lambda \in [\frac{1}{2}, 1-\delta)$ allows for a positive speed to the east. This means that there exists a deterministic vector $v = (v_x, v_y)$ with $v_x > 0$ and $v_y = 0$, such that $\mathbb{P}_0$ almost surely[3]:*

$$\frac{X_t}{t} \rightarrow_{t\rightarrow\infty} v \ .$$

Proof: The proof boils down to applying a very powerful result about RWRE's to guarantee that there exists a vector v as in the statement of the theorem (see Theorem 17). We then argue by a simple symmetry consideration that $v_y = 0$ (see Lemma 20).

Let us first comment on the spirit of the RWRE result, for walks in a d dimensional grid, with $d \geq 1$. On the line, i.e., $d = 1$, we saw that the dynamics of the walk is governed by essentially one parameter, which we coined the *governing ratio*. We will soon define an analogous ratio for the grid.

[3] This means, that on an event of $\mathbb{P}_0$ measure 1 we have the following convergence. In words, this can be phrased as "for almost all environments and walk trajectories", where the meaning of "all" is to be understood with respect to $\mathbb{P}_0$.

Theorem 17, which we will take as a black box, states that the behavior of the ratio is related to the behavior of the walk, much in the same way as on the line.

There is one difference however. On the line (under the (IID) assumption), we were able to compute the governing ratio by looking at just one point. This time the ratio is a little bit more involved, though it is still a “local” parameter. It depends on a big finite *box* (as defined later).

We next introduce the notations and definitions we need in order to state Theorem 17. By translation invariance, the behavior does not depend on the starting point, so we may assume without loss of generality, that the starting point is $0 \in \mathbb{Z}^d$.

Definition 15 (ballisticity and speed)**.** *We say that a RWRE is* ballistic *in direction* $\ell \in \mathbb{S}^d$ *if* $\mathbb{P}_0$ *a.s.*

$$\liminf_{t\to\infty} \frac{X_t \cdot \ell}{t} > 0 \ ,$$

and we say that its speed is v *if:*

$$\lim_{t\to\infty} \frac{X_t}{t} = v \ .$$

The known results on $\mathbb{Z}^d$ are criteria on the environment ensuring that the walk is ballistic. The main conditions known to imply ballisticity are known as Snitzman (T) conditions [5]. They are about the bias in the exit side of (arbitrarily large) slabs or boxes. They are also asymptotic in nature and thus hard to check. Fortunately, Snitzman was also able [7] to give a local criterion (see Definition 16) which he could connect to his other (T) conditions, hence showing that it implies ballisticity.

The local criterion is much easier to check, and this is the only result we will state[4] (see Theorem 17). In a sense, it offers an explicit connection to the one-dimensional case, and suggests that even in grids of arbitrary dimension, the walk remains essentially one-dimensional.

Assume $\ell \in \mathbb{S}^{d-1}$ is our candidate direction. Let L be a real number. For a subset $A \subseteq \mathbb{Z}^d$ we denote by T_A the hitting time of A, that is:

$$T_A := \inf\{t : X_t \in A\}.$$

We define the box $\mathcal{B}_L$ as follows

$$\mathcal{B}_L := \left\{x \in \mathbb{Z}^d : x \in -(L-2),(L+2) \times (-L,L)^{d-1})\right\}.$$

The constant 2 appearing in the previous expression is not relevant for the computations we are going to do next so we will omit it and refer to $\mathcal{B}_L$ as the cube of length L. We also define the “good exit side” as $\partial_+ B$

$$\partial_+\mathcal{B} = \{x \in \mathcal{B} : e_1 \cdot x > L,\ \forall j \in [2,d], |e_j \cdot x| \leq L\}.$$

[4]In fact we state a weaker form of the general result.

We can now define $\rho_{\mathcal{B}}(\omega)$, analogously to the $1D$ case as the probability to exit through one of the bad sides over the probability to exit through the good side. In symbols,

$$\rho_{\mathcal{B}}(\omega) := \frac{P_{\omega,0}(T_{\partial\mathcal{B}} \neq T_{\partial_+\mathcal{B}})}{P_{\omega,0}(T_{\partial\mathcal{B}} = T_{\partial_+\mathcal{B}})}.$$

Definition 16 (effective criterion). *We say that the effective criterion with respect to ℓ holds if, for some $L > c_1$ we have that*

$$\inf_L \left\{ c_2 \ln\left(\kappa^{-1}\right)^{3(d-1)} L^{4(d-1)+1} \mathbb{E}[\rho_{\mathcal{B}_L}] \right\} < 1.$$

Recall that κ is the ellipticity constant (see 2.1.1). The constant c_1 is not explicit.

Theorem 17. *The effective criterion with respect to ℓ implies the strongest of Snitzman's conditions (known as $(T') \mid \ell$) which in particular implies the walk is ballistic in direction ℓ and it has speed v with $v \cdot \ell > 0$, where $\cdot$ denotes the scalar product.*

Remark 18. *In fact, stated like this, Theorem 17 is a combination of Remark* 3.12 *Theorem* 3.17 *and Theorem* 3.26 *in [2].*

Now that we have introduced the result from RWRE theory, let us see how it applies for our particular choice of RWRE, namely the PF-NLA model. We will check the following.

Lemma 19. *For the PF-NLA model, the effective criterion, as defined in Definition 16, holds with $\ell = (1, 0)$, where λ is an arbitrary constant (that we set to $\frac{1}{2}$ for convenience), and μ is a small constant.*

Let us assume for now that the Lemma 19 holds (for conveniently chosen parameters) with correct direction being the "East". Theorem 17 implies that the rescaled walk X_t/t almost surely (with respect to the law of the environment and the walk itself) converges to a constant $v = (v_1, v_2, \ldots, v_d) \in \mathbb{R}^d$. All we need to check to complete the proof of Theorem 14 is that $v_j = 0$, for all $j \geq 2$, which we do by a symmetry argument.

Lemma 20. *Under the previous assumptions, we have that $v_j = 0$, for all $j \geq 2$ necessarily.*

Proof: [Proof of Lemma 20] To be formal, we will use a coupling argument. Let ω be an environment on the grid, we introduce its *reflection* $\tilde{\omega}$ where the transition to North and South are exchanged at each point. In symbols,

$$\begin{aligned} &\tilde{\omega}(x, x+u) = \omega(x, x+u) \text{ if } u \in \{(-1,0),(1,0)\}, \\ &\tilde{\omega}(x, x+u) = \omega(x, x-u) \text{ otherwise.} \end{aligned} \tag{7}$$

Notice that if ω has the PF-NLA law with correct direction being $(1, 0)$ (the "east"), then so does $\tilde{\omega}$ by definition.

Let $\omega, \tilde{\omega}$ be a pair of reflected environments, and let X, Y be a pair of walks, such that X follows environment ω and Y is built from X by exchanging any step $(0,1)$ by $(0,-1)$ and a step $(0,-1)$ by $(0,1)$. It can be checked using (7) that Y follows $\tilde{\omega}$.

On the one hand, we know that $\frac{X_t}{t} \to v$ and also $\frac{Y_t}{t} \to v'$ for some deterministic vectors $v, v' \in \mathbb{R}^d$. Moreover, the two walks X and Y are linked so that all coordinates of v but the first should be the opposite of v'. But since ω and $\tilde{\omega}$ both have the PF-NLA law, it must be that $v = v'$. So for $j \geq 2$, $v_j = -v_j$ and this implies that $v_j = 0$. □

Proof: [Proof of Lemma 19] We next prove that the effective criterion given by Definition 16 applies if μ is a small enough constant. For this purpose, we will show how to apply Theorem 17 for the PF-NLA model on $\mathbb{Z}^d$ [compare also [7], section 4].

Observe that the ellipticity constant is $\kappa = \frac{1-\lambda}{2d}$. To verify that the criterion of Theorem 17 is satisfied it is enough to check the following:

$$\inf_L \left(\left(\log \frac{1}{\kappa} \right)^3 L^{4d} \mathbb{E}(\rho_{\mathcal{B}}) \right) < \epsilon, \tag{8}$$

where $\varepsilon = \varepsilon(d)$ is a small dimension dependent constant.

We will ignore the $+2$ and -2 in the definition of $\mathcal{B}$ and also set $\tilde{L} = L$, so we will refer to the box $\mathcal{B}$ as the *cube of length* L.

$$\mathcal{B}_L := [-L, L]^d.$$

We will show that for *some* choice of L and if μ is a small enough constant (dependent on d), then Eq. 8 holds.

In order to check Criterion (8), we start by a standard fact about random walks in $\mathbb{Z}$.

Lemma 21. *Let Y_t be a simple possibly lazy, unbiased random walk on the line. Then, for any $\alpha > 0$ there exists a constant $c(\alpha) = c > 0$ (which doesn't depend on the laziness parameter) such that, if t is big enough,*

$$\mathbb{P}\left(\sup_{0 \leq s \leq t} |Y_s| \geq \alpha t \right) \leq \exp(-ct).$$

Proof: By a comparison argument, it is enough to check the statement if Y is not lazy. A standard fact relates the distribution of the maximum at time t to the position at time t as follows

$$\mathbb{P}(\sup_{0 \leq s \leq t} |Y_s| \geq \alpha t) = 2\left[\mathbb{P}(Y_t = \alpha t) + 2\mathbb{P}(Y_t > \alpha t)\right] \leq 6\mathbb{P}(Y_t \geq \alpha t).$$

The term appearing on the right hand side is bounded by e^{-ct}, if t is big enough using the Chernoff bound. □

We now proceed to checking that Equation (8) holds. We will distinguish two cases. The first is when there are no mistakes inside the box we are considering.

Lemma 22 (No mistakes in the cube). *If there are no mistakes inside the cube $\mathcal{B}_\mathcal{L}$ of length L, then $\rho_{\mathcal{B}_\mathcal{L}} \leq 2d \cdot e^{-cL}$, for some $c > 0$.*

Proof: Remember that we set $\lambda = \frac{1}{2}$. Consider the PF-NLA walk X induced by the "no mistakes" environment in the cube $\mathcal{B}_\mathcal{L}$. Namely, at any step X jumps to the right with probability $\lambda + (1-\lambda)/2d$ and to any other point with probability $(1-\lambda)/2d$. It is then easy to check that, for any $j \in [d]$, $(X_t - \lambda e_1) \cdot e_j$ is a simple lazy random walk on the line (we denote by $\cdot$ the scalar product). Hence, for any $j \in [d]$, Lemma 21 yields

$$\mathbb{P}\left(\sup_{0\leq s\leq 3L} |(X_s - \lambda e_1) \cdot e_j| \geq 0.3L\right) \leq \exp(-cL).$$

Using a union bound, we get

$$\mathbb{P}\left(\forall j \in [d], \sup_{0\leq s\leq 3L} |(X_s - s/2e_1) \cdot e_j| \geq 0.3L\right) \leq d\exp(-cL).$$

In particular, under the complementary of the event appearing in the left hand side, the walk exits through the correct side. Indeed at time $3L$, $X_{3L} \cdot e_1$ is guaranteed to be in $[3\lambda L - 0.3L, 3\lambda L + 0.3L]$ which is outside of the cube, since we picked $\lambda \geq 1/2$. Also, for any $j \geq 2$ and any time $s \leq 3L$, $|X_s \cdot e_j|$ is never bigger than $0.3L < L$. This implies that the walk exited through the east necessarily.

Thus we can bound the ratio $\rho_{\mathcal{B}_\mathcal{L}}$ as follows $\rho_{\mathcal{B}_\mathcal{L}} \leq \frac{de^{-cL}}{1-de^{-cL}} \leq 2de^{-cL}$. □

Lemma 22 gives a bound on ρ when the advice has no mistakes inside $\mathcal{B}$. The following observation, which is a very crude bound, settles all other cases. Remember that we denote by κ the ellipticity constant defined in Section 2.1.1. It is the smallest transition probability in any direction for any point and any environment, $\mathbb{P}$ almost surely. In our case it is $\frac{1-\lambda}{2d}$.

Lemma 23 (Worst-case for the advice configuration inside $\mathcal{B}_L$). *No matter what the advice is in $\mathcal{B}$, the ratio is always at most $\rho_{\mathcal{B}_\mathcal{L}} \leq \kappa^{-L} = (\frac{2d}{1-\lambda})^L$.*

Proof: Indeed, it is always possible to exit by going straight to the correct side, in L steps. Each step has probability at least $\kappa = \frac{1-\lambda}{2d}$. □

To conclude it only remains to estimate $\mathbb{E}\rho_{\mathcal{B}_\mathcal{L}}$. The probability that there are no mistakes in the cube $\mathcal{B}_\mathcal{L}$ is $(1-\mu)^{(2L)^d}$. We thus obtain

$$\mathbb{E}\rho_{\mathcal{B}_\mathcal{L}} \leq 2d\exp(-cL) + \left(\frac{2d}{1-\lambda}\right)^L (1-(1-\mu)^{(2L)^d}). \tag{9}$$

Recall that $\lambda \in (\frac{1}{2}, 1-\delta)$. Remember that all we need to do is find an L so that for μ small enough

$$\left(\log\frac{1}{\kappa}\right)^3 L^{4d}\mathbb{E}\rho_{\mathcal{B}_\mathcal{L}} \leq \varepsilon,$$

for some small constant $\varepsilon > 0$. Remember that $\frac{1}{\kappa} = \frac{2d}{1-\lambda} \leq \frac{2d}{\delta}$. From Equation 9, we know it is enough to show that for *one* choice of parameters L, μ we have,

$$\left(\log \frac{1}{\kappa}\right)^3 L^{4d} \left(2d \exp(-cL) + \left(\frac{2d}{1-\lambda}\right)^L (1 - (1-\mu)^{(2L)^d})\right) \leq \varepsilon.$$

□

The function $L \mapsto L^C \exp(-cL)$ goes to 0 as X goes to infinity, so it is possible to choose $L = L_0$ big enough so that

$$\left(\log \frac{2d}{\delta}\right)^3 L^{4d} \left(2d \exp(-cL)\right) \leq \varepsilon/2,$$

and then pick μ small enough (this depends on the parameter δ), such that

$$\left(\log \frac{2d}{\delta}\right)^3 L_0^{4d} \left(\frac{2d}{\delta}\right)^{L_0} (1 - (1-\mu)^{(2L_0)^d}) \leq \varepsilon/2.$$

□

Remark 24. *The question whether results similar to those we presented on the line, i.e. estimates on the hitting time of a designated target under the PF-NLA model, can be obtained for general (finite) graphs seems to be beyond the scope of currently known techniques. In particular, arbitrary finite graphs lack the symmetry of the grid making it unclear how to use renormalization arguments (i.e. induction) such as in the proof of Theorem 17. Establishing such results for general graphs remains open for further theoretical study.*

5 Quantitative comparison to experimental measurements

Using the long obstacle experiments as depicted in figure 5A-B of the main text, we found that the ant-team turns to move against the direction of the nearby scent marks once every 37.4 cm. In our theoretical model, a step corresponds to the persistence length of motion. Denoting this persistence by ℓ (measured in cm) implies turning against the advice once per $\frac{37.4}{\ell}$ steps. This translates to a following probability of listening $\lambda = 1 - \frac{\ell}{37.4}$.

In the case of the modified obstacle in which all scent marks are misleading the carrying-team requires exponential time to distance itself from the slit (see figure 5C of the main text). Experimentally, we found that, in this case, first passage times to distance d scales as $e^{0.33D}$. Descretizing D into units of the persistence length ℓ we obtain a passage time that is proportional to $e^{0.33 \cdot \ell \cdot \frac{D}{\ell}} = (e^{0.33\ell})^d$, where d is the distance with step units. Using the equation in the second bullet of Lemma 9, this scaling corresponds to

$$\alpha^d = E(\rho)^d = \left(\frac{1+\lambda}{1-\lambda}\right)^d$$

where the equality $E(\rho) = \frac{1+\lambda}{1-\lambda}$ follows from the fact that $\mu = 1$ (all advice is misleading), and using Equation 6.

In order for these two different measurements to agree the following equations have to simultaneously satisfied:

$$\frac{1+\lambda}{1-\lambda} = e^{0.33\ell} \quad \text{and} \quad \lambda = 1 - \frac{\ell}{37.4}$$

Plugging the second equation into the first yields persistence length $\ell = 6.9cm$ and a listening probability $\lambda = 0.81$.

References

[1] David Aldous and James Allen Fill. Reversible markov chains and random walks on graphs, 2002. Unfinished monograph, recompiled 2014.

[2] Alexander Drewitz and Alejandro F. Ramiréz. Selected topics in random walk in random environment. *Topics in Percolative and Disordered Systems, Springer Proceedings in Mathematics and Statistics*, 69:23–83, 2014.

[3] Nicolas Hanusse, David Ilcinkas, Adrian Kosowski, and Nicolas Nisse. Locating a target with an agent guided by unreliable local advice: How to beat the random walk when you have a clock? In *Proceedings of the 29th ACM SIGACT-SIGOPS Symposium on Principles of Distributed Computing*, PODC '10, 2010.

[4] Nicolas Hanusse, Dimitris Kavvadias, Evangelos Kranakis, and Danny Krizanc. Memoryless search algorithms in a network with faulty advice. *Theoretical Computer Science*, 402(2,3):190 – 198, 2008.

[5] Alain-Sol Snitzman. Topics in random walks in random environment. *ICTP Lecture Notes Series*, 2004.

[6] Fred Solomon. Random walks in a random environment. *Ann. Probab.*, 3(1):1–31, 02 1975.

[7] Alain-Sol Sznitman. An effective criterion for ballistic behavior of random walks in random environment. *Probability Theory and Related Fields*, 122(4):509–544, 2002.
